# Validity and Reliability of the European Portuguese Version of Neuropsychiatric Inventory in an Institutionalized Sample

**DOI:** 10.14740/jocmr1959w

**Published:** 2014-10-16

**Authors:** Ana Rita Ferreira, Sonia Martins, Orquidea Ribeiro, Lia Fernandes

**Affiliations:** a Faculty of Medicine, University of Porto, Al. Hernani Monteiro, 4200-319 Porto, Portugal; b Research and Education Unit on Aging (UNIFAI), University of Porto, Rua Jorge de Viterbo Ferreira, no. 228, 4050-313 Porto, Portugal; c Department of Health Information and Decision Sciences (CIDES) and CINTESIS, Faculty of Medicine, University of Porto, Al. Hernani Monteiro, 4200-319 Porto, Portugal; d UNIFAI/CINTESIS Research Unit, Faculty of Medicine, University of Porto. Clinic of Psychiatry and Mental Health, CHSJ, Porto, Al. Hernani Monteiro, 4200-319 Porto, Portugal

**Keywords:** Behavior disorders, Dementia, Aged, Neuropsychiatric inventory, Validation study

## Abstract

**Background:**

Neuropsychiatric symptoms are very common in dementia and have been associated with patient and caregiver distress, increased risk of institutionalization and higher costs of care. In this context, the neuropsychiatric inventory (NPI) is the most widely used comprehensive tool designed to measure neuropsychiatric Symptoms in geriatric patients with dementia. The aim of this study was to present the validity and reliability of the European Portuguese version of NPI.

**Methods:**

A cross-sectional study was carried out with a convenience sample of institutionalized patients (≥ 50 years old) in three nursing homes in Portugal. All patients were also assessed with mini-mental state examination (MMSE) (cognition), geriatric depression scale (GDS) (depression) and adults and older adults functional assessment inventory (IAFAI) (functionality). NPI was administered to a formal caregiver, usually from the clinical staff. Inter-rater and test-retest reliability were assessed in a subsample of 25 randomly selected subjects.

**Results:**

The sample included 166 elderly, with a mean age of 80.9 (standard deviation: 10.2) years. Three out of the NPI behavioral items had negative correlations with MMSE: delusions (r_s_ = -0.177, P = 0.024), disinhibition (r_s_ = -0.174, P = 0.026) and aberrant motor activity (r_s_ = -0.182, P = 0.020). The NPI subsection of depression/dysphoria correlated positively with GDS total score (r_s_ = 0.166, P = 0.038). NPI showed good internal consistency (overall α = 0.766; frequency α = 0.737; severity α = 0.734). The inter-rater reliability was excellent (intraclass correlation coefficient (ICC): 1.00, 95% confidence interval (CI) 1.00 - 1.00), as well as test-retest reliability (ICC: 0.91, 95% CI 0.80 - 0.96).

**Conclusion:**

The results found for convergent validity, inter-rater and test-retest reliability, showed that this version appears to be a valid and reliable instrument for evaluation of neuropsychiatric symptoms in institutionalized elderly.

## Introduction

Since its first formal definition, Alzheimer’s disease (AD) has also included non-cognitive symptoms such as delusions, misidentification, hallucinations, anxiety, and apathy [1, 2]. However, behavioral and psychological symptoms were excluded from the main international classifications and diagnostic criteria, playing a secondary role within the diagnosis of dementia. Later, in 1996, those non-cognitive manifestations of dementia were defined by the International Psychogeriatric Association as “signs and symptoms of disturbed perception, thought, content, mood, or behavior that frequently occur in patients with dementia.” [3, 4] and a large number of population-based studies confirmed the presence of this heterogeneous group of symptoms and behaviors.

The near universal prevalence of behavioral and psychological symptoms of dementia (BPSD) in all dementia patients [5-8] at any disease stage and regardless of its etiology [9] has focused attention on reliable and valid measures of BPSD and effective and safe available treatments. According to Frisoni et al [10], 50% of patients with dementia have at least four BPSD simultaneously and its prevalence is likely to increase during the natural course of dementia [11] combined with seriously disabling effects on patients and caregivers.

The prevalence rates of BPSD largely depend on the type of the sample and the setting considered, being less frequent and severe in community-dwelling subjects with dementia than in hospitalized and institutionalized patients [12]. However, even in mildly impaired community-dwelling samples, the BPSD contribute significantly to the burden of dementia care [13]. Due to their high occurrence, BPSD have become a relevant and significant clinical and research targets [14-16].

BPSD have been referred as highly correlated with high levels of distress and caregiver burden, as well as with increased use of health care resources, risk of institutionalization [3, 17, 18] and mortality [19]. Additionally, approximately 50% of subjects with mild cognitive impairment (MCI) and 25% of those with normal cognition present at least one BPSD [20], suggesting that they can include early signs of dementia preceding the onset of cognitive deficits [21-23].

Taking this into consideration, the early recognition and accurate diagnosis of BPSD, based on valid and reliable assessment tools, appears as an important feature for its optimal treatment, contributing to decrease of associated adverse outcomes. BPSD assessment has been highly recognized as crucial to the management of dementia, and it has become an important aspect of dementia research worldwide [9].

In this context, the neuropsychiatric inventory (NPI) [24] is the most widely comprehensive tool used to assess behavioral and psychological disturbances, based on an informant interview format rating scale. The NPI has been translated and validated into a number of languages and is widely accepted as the standard measure of BPSD in clinical trials and in several population-based studies into dementia and related disorders [20, 25, 26]. This inventory has also been used as an outcome measure in various other research studies, for example to gather information that distinguishes among etiologies of dementia [24, 27], to measure longitudinal changes in BPSD in elderly nursing home residents [28] and to assess the effects of pharmacological treatments [29].

In the original studies [24, 30], the NPI showed good reliability and validity. Inter-rater reliability ranged from 93.6% to 100%, according to the subdomain considered, and test-retest reliability was also shown to be very high. Content validity was high, as rated by internationally recognized experts in geriatric psychiatry. As a whole, those results proved its robustness as a tool to assess BPSD across a variety of settings [26].

Although Portugal has one of the highest rates of mental disorders in Europe [31], little is known about BPSD prevalence and validated measures to achieve this goal are lacking.

The aim of this study was to contribute to the further understanding of the psychometric properties of the European Portuguese version of NPI, by presenting the preliminary results of its validation study in a geriatric nursing home sample.

## Material and Methods

### Sample and procedures

A cross-sectional study was conducted with a convenience sample of institutionalized patients (≥ 50 years old) in three nursing homes in north of Portugal, between September 2012 and April 2013. All permanently institutionalized subjects were eligible for the study. However, subjects with delirium, those who were terminally ill, unable to speak Portuguese or who refused to participate were not included. The presence of BPSD was not considered to be a prerequisite to the inclusion in the study.

All subjects included were assessed with the following instruments: mini-mental state examination (MMSE) [32, 33], as a cognitive global measure, adults and older adults functional assessment inventory (IAFAI) [34], for functional assessment, and geriatric depression scale (GDS) [35, 36], as a measure of depressive symptomatology. The NPI was administered to a formal caregiver, usually from the clinical staff, for BPSD assessment. In order to fit the assessment protocol to the context of the investigation, previously validated versions of the instruments or versions adapted for the Portuguese population were used. In this study, the European Portuguese translation of NPI by Leitao and Nina [37] was used.

Complete demographic and clinical characteristics of the sample were obtained through chart review.

Regarding the inter-rater and test-retest reliability analysis, it was carried out in a sample of 25 randomly selected elderly people, recruited only from one of the nursing homes. In the inter-rater reliability process, two researchers completed the NPI independently and the interviewers were alternated. The test-retest reliability was determined by conducting a second NPI interview within 2 weeks.

All procedures concerning ethical approval were obtained from the board of the institutions where the study was held. All the necessary measures to safeguard participants’ anonymity and confidentiality of information were also thoroughly followed. Informed consent was obtained from the patient and/or from their legal representative.

### Description of the instrument

NPI is an informant-based rating scale developed to assess neuropsychiatric symptoms in dementia. The instrument consists of 12 items, with each of them evaluating 12 common BPSD, including delusions, hallucinations, agitation/aggression, depression, anxiety, elation, apathy, disinhibition, irritability, aberrant motor behavior, and sleeping and eating disorders [24, 30]. The questions are approached in a comprehensive and time-efficient manner through the use of screening questions for each target behavior. If the behavior is absent, no further questions are needed; if present, more comprehensive information is obtained by a series of in-depth questions to probe specific symptomatology, its frequency (on a scale from 1 to 3) and severity (on a scale from 1 to 4).

The NPI total or composite score is calculated by multiplying the frequency and severity rates per domain (maximum score per domain is 12) and adding them up (minimum is 0 and maximum 144). Moreover, the same interview can register caregiver emotional distress for each item and for the whole scale [38].

### Statistical analysis

Statistical analyses were performed using the Statistical Package for the Social Sciences Version 21.0 for Windows software (SPSS). Patient characteristics are presented as raw frequencies and percentages for categorical variables and as mean and standard deviation (SD) for continuous variables, when normality could be assumed and median, minimum, maximum or percentiles 5 and 95, otherwise. For comparing the distribution of the total NPI between two or more independent groups, Mann-Whitney or Kruskal-Wallis tests were used for continuous variables when normality could not be assumed. Convergent validity was explored by calculating Spearman’s p rank correlation coefficients between NPI domains and total scores for MMSE, GDS and IAFAI, at a significance level of 0.05. Cronbach’s coefficient alpha (α) was determined to assess internal consistency. Inter-rater and test-retest reliability were assessed by calculating the intraclass correlation coefficient (ICC) and 95% confidence intervals (CIs).

## Results

In the present study, a total of 166 participants was assessed. All participants lived in long-term care institutions. The main socio-demographic and clinical characteristics of the participants are presented in detail in Table 1.

**Table 1:** Demographic and Clinical Characteristics of Sample

n = 166	
Age (years) mean (SD)	80.9 (10.2)
Gender, n (%)	
Male	16 (10)
Female	150 (90)
Marital status, n (%)	
Single	51 (31)
Married	12 (7)
Divorced/separated	17 (11)
Widowed	86 (51)
Education, n (%) (n = 164)	
< 4 years	88 (53)
4 years	53 (33)
> 4 years	23 (15)
Institutionalization, n (%)	
0 - 4 years	110 (64)
5 - 9 years	25 (15)
10 - 14 years	16 (11)
≥ 15 years	15 (10)
MMSE, n (%) (n = 163)	
No cognitive deficit	66 (40.5)
Cognitive deficit	97 (59.5)
GDS, n (%) (n = 158)	
No depression	85 (54)
Depression	73 (46)
IAFAI, mean (SD)	
Total disability	47 (25)

The average NPI-total score for this sample was 6 (SD: 12), with a minimum of 0 and a maximum of 76. The most frequent neuropsychiatric domains were: sleep and nighttime behavior disorders (54%), delusions (22%), depression/dysphoria (19%), irritability/lability (17%) and agitation/aggression (15%) (Table 2). No significant correlations between NPI (domains and total) and age, gender and education level of the elderly were found.

**Table 2:** Frequency of NPI Domains

NPI domains	Not present n (%)	Present n (%)
Delusions	130 (78)	36 (22)
Hallucinations	154 (93)	12 (7)
Agitation/aggression	141 (85)	25 (15)
Depression/dysphoria	134 (81)	32 (19)
Anxiety	149 (90)	17 (10)
Elation/euphoria	161 (97)	5 (3)
Apathy/indifference	154 (93)	12 (7)
Disinhibition	157 (95)	9 (5)
Irritability/lability	137 (83)	29 (17)
Aberrant motor behavior	159 (96)	7 (4)
Sleep/nighttime behavior disorders	77 (46)	89 (54)
Appetite and eating changes	155 (93)	11 (7)

As expected a significant difference regarding total score of NPI (P = 0.007) was found, for higher values in the group with cognitive deficit, assessed by MMSE (mean rank 89.66 vs. 70.73).

Three out of the NPI behavioral items (frequency × severity) had significant negative correlations with MMSE: delusions (r_s_ = -0.177, P = 0.024), disinhibition (r_s_ = -0.174, P = 0.026) and aberrant motor activity (r_s_ = -0.182, P = 0.020). The NPI domain of depression/dysphoria correlated positively with GDS total score (r_s_ = 0.166, P = 0.038) (Table 3). A positive correlation between the total score of NPI and the cognitive domain of IAFAI (r_s_ = 0.258; P = 0.002) was also found.

**Table 3:** Correlations Between NPI, MMSE and GDS

NPI domains (FxS)	MMSE r_s_ (P)	GDS r_s_ (P)
Delusions	-0.177*(0.024)	0.126 (0.114)
Hallucinations	-0.060 (0.443)	0.074 (0.357)
Agitation/aggression	-0.090 (0.252)	-0.014 (0.857)
Depression/dysphoria	-0.010 (0.901)	0.166* (0.038)
Anxiety	0.007 (0.928)	0.027 (0.736)
Elation/euphoria	-0.076 (0.337)	-0.104 (0.192)
Apathy/indifference	-0.118 (0.133)	0.081 (0.309)
Disinhibition	-0.174*(0.026)	0.006 (0.943)
Irritability/lability	0.079 (0.316)	-0.037 (0.649)
Aberrant motor behavior	-0.182*(0.02)	-0.013 (0.873)
Total	-0.132 (0.092)	0.115 (0.151)
Sleep/nighttime behavior disorders	0.058 (0.465)	0.069 (0.391)
Appetite and eating changes	0.016 (0.839)	0.047 (0.557)

Spearman test; *Correlation is significant at the 0.05 level.

The NPI showed good internal consistency (overall α = 0.766; frequency α = 0.737; severity α = 0.734; distress α = 0.755). Cronbach’s alpha varied between 0.706 and 0.767 for product of frequency and severity; 0.673 - 0.738 for frequency, 0.676 - 0.735 for severity and 0.692 - 0.757 for distress in individual items (Table 4).

**Table 4:** Cronbach’s Alpha (α) for Individual Domains of NPI

NPI domains	FxS (α)	Frequency (α)	Severity (α)	Distress (α)
Delusions	0.727	0.697	0.690	0.721
Hallucinations	0.718	0.680	0.681	0.705
Agitation/aggression	0.706	0.673	0.676	0.692
Depression/dysphoria	0.753	0.735	0.728	0.737
Anxiety	0.765	0.738	0.728	0.757
Elation/euphoria	0.767	0.733	0.735	0.746
Apathy/indifference	0.757	0.733	0.724	0.740
Disinhibition	0.756	0.716	0.716	0.724
Irritability/lability	0.732	0.704	0.703	0.754
Aberrant motor behavior	0.765	0.732	0.732	0.749
Total	0.766	0.737	0.734	0.755

To analyze the inter-rater and test-retest reliability, a subsample of 25 elderly, with a mean age of 81.6 (SD: 9.2), mostly female (76%), widowed (64%) and with lower educational level (92% ≤ 4 years) was used.

The inter-rater reliability was excellent for total (ICC: 1.00, 95% CI: 1.00 - 1.00) and for all domains of NPI (ICC: 1.00, 95% CI: 1.00 - 1.00).

The test-retest reliability was excellent for total (FxS ICC: 0.909, 95% CI: 0.80 - 0.96; distress ICC: 0.89, 95% CI: 0.766 - 0.950). The results for each NPI domains are presented in Table 5.

**Table 5:** Test-Retest Reliability

NPI domains	FxS ICC (95% CI)	Distress ICC (95% CI)
Delusions	0.936 (0.860, 0.971)	0.958 (0.908, 0.981)
Hallucinations	0.897 (0.780, 0.953)	0.882 (0.750, 0.946)
Agitation/aggression	0.799 (0.596, 0.906)	0.818 (0.630, 0.916)
Depression/dysphoria	0.728 (0.473, 0.870)	0.699 (0.427, 0.855)
Anxiety	0.670 (0.381, 0.840)	0.508 (0.149, 0.749)
Elation/euphoria	0.600 (0.276, 0.802)	0.923 (0.834, 0.965)
Apathy/indifference	0.691 (0.415, 0.851)	0.621 (0.307, 0.813)
Disinhibition	0.296 (-0.104, 0.614)	0.531 (0.179, 0.762)
Irritability/lability	0.524 (0.170, 0.758)	0.542 (0.195, 0.768)
Aberrant motor behavior	0.976 (0.946, 0.989)	1.000 (1.000, 1.000)
Total	0.909 (0.804, 0.959)	0.890 (0.766, 0.950)
Sleep/nighttime behavior disorders	0.650 (0.350, 0.829)	0.741 (0.496, 0.877)
Appetite and eating changes	0.607 (0.286, 0.805)	0.648 (0.347, 0.828)

ICC: intraclass correlation coefficient; CI: confidence interval.

## Discussion

This study indicates that the European Portuguese version of NPI has good validity and reliability, presents significant convergent validity, as well as robust positive data on inter-rater and test-retest reliability.

In this study, the most frequent symptoms were the same found in previous studies [39-44], in particularly depression. Major depressive disorder, minor depression, dysthymia or subsyndromal, but still clinically significant depressive symptoms not only are common in the long term care facilities, but also appear to be common comorbid conditions with dementia. However, the most frequent behavioral symptom was related to sleep/nighttime domain. Several factors can contribute to sleep impairment. It is well known that age itself can induce alteration in sleep patterns [45] just like the medical conditions and medications. But sleep problems tend to be more common and severe in nursing home residents, which would be expected based on increase of age. Among the major factors, one can enumerate the nursing home environmental factors or the behavioral lifestyle associated with routines that conduct to inactivity during daylight hours [46]. Another explanation can be the possible association of poor quality of sleep with the prevalence of depression and depressive symptoms found in this sample.

Apathy also appears as one of the most frequent domains in these previous studies, but not in the present study. This can be explained in part by the fact that some of these studies included only patients with dementia [39, 40, 42] with the correlated potential for referral bias leading to higher frequency estimates in contrast with the present study that addressed both demented and healthy subjects which can explain the minor prevalence of this particular BPSD. On the other hand, clinicians often regard depression as a model for defining apathy, even if these concepts have to be distinguished.

The least common domains, namely euphoria, aberrant motor behavior, disinhibition and hallucinations, were similar to those found in previous studies [39, 42, 43]. In fact, neuropsychiatric symptoms are ubiquitous in nursing home demented patients, with prevalence rates above 80% [47] and the institutionalization must be taken into account as an important environmental factor.

Moreover, no significant correlations between the NPI (total and domains) with age, gender or education level of the elderly were found, which can be explained by the fact that neuropsychiatric symptoms might be independent of demographic variables. Some studies had already found this lack of correlation [40], but others found some correlation. For example, Mega et al [17] have demonstrated a significant association between male patients and agitation, and Cummings et al [24] have shown a significant relationship between apathy and age, with older patients being rated as more apathetic. Zuidema et al [48] also found that depression and anxiety were more frequent in female patients, and apathy in male institutionalized patients.

As in other studies [48], BPSD were associated with cognitive decline, with most symptoms occurring in those who showed decline.

The psychometric proprieties of the European Portuguese version of NPI seem to be in accordance with the original studies [24, 30], as well as with other previous international validation studies [39-44, 49-51].

This version also showed good convergent validity, corroborated by the correlations found with other measures. As in the original study [24], the same NPI domains (except for anxiety) were negatively correlated with MMSE. Moreover, a positive correlation between depression/dysphoria NPI domain and a depression assessment instrument (in the present study, the GDS) was also found in other previous studies [24, 49].

Regarding the inter-rater reliability, the values for total and for all domains of NPI, were higher than in other studies [43], with 100% of concordance between the two raters. This analysis demonstrates that this version is highly reliable when used by different examiners.

The previous validations studies also showed good test-retest correlations coefficients, ranging between 0.63 and 0.96 [24, 40, 50, 51]. In the present study, the test-retest reliability was assessed using ICC, presenting globally good values for total and for all domains, except for disinhibition and irritability/lability. These lowest values may reflect more variability in these behaviors or more difficulty for caregivers in reliably assessing and reporting these BPSD in response to NPI questions.

There is some heterogeneity in the methodological issues in the previous validation studies that need to be taken into consideration, such as the setting (outpatient vs. institutionalization), sample (patients only with dementia vs. patients without dementia) and statistical procedures. On the other hand, the differences in the results from the present study and those conducted in other countries may be related to different backgrounds, and differences in understanding and rating of the symptoms.

The strengths of this study were that, to our knowledge, this is the first validation study of the European Portuguese version of the NPI. Moreover, this validation process could also provide specific information regarding the neuropsychiatric symptoms of a sample of Portuguese elderly people. Finally, in the present study elderly with and without cognitive deficit were included, as well as patients with previous major psychiatric disorders (e.g. depression and schizophrenia), in some cases with a concurrent diagnosis of dementia.

This study presents some limitations. First, detailed information concerning some clinical characteristics (e.g. type of dementia) was not available. Second, pharmacological treatment was not controlled, so some substances could have induced, alleviated or eliminated some BPSD. Third, as this is a three-site study, validation of the performance of NPI will be required in other settings to assure generalizability. Further research should focus on the use of this instrument in various settings by different levels of health professionals.

In conclusion, the European Portuguese version of NPI showed a good internal consistency, convergent validity, as well as an excellent inter-rater and test-retest reliability. These results suggested that this version emerges as a promising tool in the evaluation of neuropsychiatric symptoms in institutionalized elderly people.

However, additional research is recommended with samples recruited from other settings for further validation of these results, to analyze other psychometric proprieties of the present version.
